# Supplementary material for: Influence of Previous COVID-19 and Mastitis Infections on the Secretion of Brain-Derived Neurotrophic Factor and Nerve Growth Factor in Human Milk
Source: Int J Mol Sci. 2021 Apr 8;22(8):3846. doi: 10.3390/ijms22083846 (PMC8068104; doi:10.3390/ijms22083846)
Supplement: Supplementary file 1 [file ijms-22-03846-s001.pdf]

Table S1. Participant characteristics[1] with a confirmed COVID-19 PCR test (PCR-M1 to PCR-M12) or with viral symptoms suggestive of COVID-19 (Viral-M1 to Viral-M13) without PCR testing.

Mothers	Confirmed RNA-SARS-CoV-2/PCR	Symptoms of viral infection	Time from infection to collection (mo)	Infant gender	Lactation time (months)	Maternal age
**PCR-M1**	+COVID19-PCR	Fever, fatigue, cough	4.1	Female	5	36
**PCR-M2**	+COVID-19-PCR	NA	1.8	Female	6	26
**PCR-M3**	+COVID-19-PCR	NA	1.7	Female	6	26
**PCR-M4**	+COVID-19-PCR	NA	0.9	Male	5	33
**PCR-M5**	+COVID-19-PCR	NA	0.5	Male	8	37
**PCR-M6**	+COVID-19-PCR	Loss of smell/taste, fever, headache	0.5	Male	10	37
**PCR-M7**	+ COVID-19-PCR	NA	1.3	Male	6	33
**PCR-M8**	+COVID-19-PCR	NA	3.7	Female	6	26
**PCR-M9**	+COVID-19-PCR	Fever, fatigue, cough	1.3	Female	4	30
**PCR-M10**	+COVID-19-PCR	headache, cough, fatigue, fever, loss of taste/smell	0.6	Female	10	27
**PCR-M11**	+COVID-19-PCR	headache, loss of smell, fever	0.8	Male	4	33
**PCR-M12**	+COVID-19-PCR	headache, loss of smell, fever	1	Male	4	32
**Viral-M1**	Absence of PCR-testing	Fever, chills, cough, body aches, nasal congestion	2.9	Female	6	32

**Viral-M2**	Absence of PCR-testing	Fever, fatigue, and cough	1.7	Female	4	30
**Viral-M3**	Absence of PCR-testing	Fever and cough	3.8	Female	5	29
**Viral-M4**	Absence of PCR-testing	Extreme fatigue, fever, nasal congestion, cough	3.3	Female	5	40
**Viral-M5**	Absence of PCR-testing	Fever, cough, some nasal congestion, headache	NA	Female	5	30
**Viral-M6**	Absence of PCR-testing	Body aches and respiratory infection	NA	Male	NA	NA
**Viral-M7**	Absence of PCR-testing	Cough lasted 4+ weeks, fever, body aches	3.4	Male	5	33
**Viral-M8**	Absence of PCR-testing	Severe respiratory infection, cough for 6 weeks, low fever	2.3	Male	5	32
**Viral-M9**	Absence of PCR-testing	Sneezing, headache, fever, vomit, runny nose	3.9	Female	5.5	23
**Viral-M10**	Absence of PCR-testing	Fever	3.3	Male	9	40
**Viral-M11**	Absence of PCR-testing	Nasal congestion	3.2	Male	8	33
**Viral-M12**	Absence of PCR-testing	Nasal congestion and fever	2.6	Female	8	34
**Viral-M13**	Absence of PCR-testing	fever, headache, nasal congestion, loss of smell/taste	2.8	Female	4	30

[1] Women did not have medical history, medication, and systematic diseases
